# Micro/Nanostructures and Mechanical Properties of Trabecular Bone in Ovariectomized Rats

**DOI:** 10.1155/2015/252503

**Published:** 2015-07-27

**Authors:** Shidi Hu, Jin Li, Lu Liu, Ruchun Dai, Zhifeng Sheng, Xianping Wu, Xiqiao Feng, Xuefeng Yao, Eryuan Liao, Evan Keller, Yebin Jiang

**Affiliations:** ^1^Institute of Metabolism and Endocrinology, The Second Xiangya Hospital, Central South University, Changsha, Hunan 410011, China; ^2^AML, Department of Engineering Mechanics, Tsinghua University, Beijing 100084, China; ^3^Comprehensive Cancer Center and Urology, University of Michigan, E Medical Center Drive, Ann Arbor, MI 48109, USA; ^4^Research & Development and Radiology, VA Southern Nevada Healthcare System, 6900 N. Pecos Road, North Las Vegas, NV 89086, USA

## Abstract

Bone mechanical properties encompass both geometric and material factors, while the effects of estrogen deficiency on the material and structural characteristics of bone at micro- to nanoscales are still obscure. We performed a series of combined methodological experiments, including nanoindentation assessment of intrinsic material properties, atomic force microscopy (AFM) characterization of trabecular (Tb) nanostructure, and Tb microarchitecture and 2D BMD. At 15 weeks after surgery, we found significantly less Tb bone mineral density (BMD) at organ (−27%) and at tissue level (−12%), Tb bone volume fraction (−29%), Tb thickness (−14%), and Tb number (−17%) in ovariectomy (OVX) rats than in sham operated (SHAM) rats, while the structure model index (+91%) and Tb separation (+19%) became significantly greater. AFM images showed lower roughness Tb surfaces with loosely packed large nodular structures and less compacted interfibrillar space in OVX than in SHAM. However, no statistically significant changes were in the Tb intrinsic material properties—nanoindentation hardness, elastic modulus, and plastic deformation—nanoindentation depths, and residual areas. Therefore, estrogen deprivation results in a dramatic deterioration in Tb micro/nanoarchitectures, 3D volumetric BMD at both organ and tissue levels, and 2D BMD, but not in the nanomechanical properties of the trabeculae per se.

## 1. Introduction

Bone strength encompasses architectural, geometric, and material factors that contribute to bone fragility [1–5]. The contributions of bone mass and bone mineral density (BMD) and architecture to bone strength are well recognized. However, these variables cannot explain all the variance of bone strength [2]. Bone is a natural material displaying a remarkable hierarchical organization, where mechanical integrity of bone is dictated by its structures and materials at different length scales, from nanoscale, for example, mineral crystals and collagen fibrils, and microscale, for example, microarchitecture and osteocyte lacunar network, to macroscale, for example, bone size and geometry [6–8]. Therefore, evaluation of the structure-function relationship between bone composition and mechanical properties at various length-scales is important to understand the bone fragility in osteoporosis.

Estrogen-deficiency attributable to menopause is considered to be a culprit of postmenopausal osteoporosis and can result in low bone mass and microarchitectural deterioration with a consequent susceptibility to fracture [9, 10]. These changes are believed to result in bone tissue loss and decrease in BMD, mainly through alterations in bone remodeling rates. The enhanced remodeling activity [11] affects bone mineralization, which in turn contributes to the degradation of microarchitecture and affects the bone strength at the macroscale [12]. Bone matrix, especially collagen cross-links, is deteriorated in postmenopausal osteoporotic individuals [13, 14]. Because mineralization significantly affects Young's modulus and hardness [15], newly formed bone would be undermineralized and less resistant to bend [16]. It might be assumed that bone material properties at the tissue level should also be impaired in estrogen deprivation that increases bone turnover. However, the material properties of bone at the micro- to nanoscales and their effects on mechanical performance are still elusive.

Nanoindentation is a widely used technique to determine the mechanical properties of bone at nanoscale [17–19]. In nanoindentation, one can extract the hardness and elastic modulus from the load-displacement curves of the measured materials, for example, osteons, lamellae and individual trabeculae, collagen fibrils, and individual mineral crystals [20–23]. The nanoindentation technique has been used to investigate the mechanical properties of various microstructures in bone with individual, age-related structural and mechanical alteration at the bone material level and the mechanical properties of bone in individuals with a particular bone disease [18, 19, 24]. Nevertheless, there is still a lack of experimental studies on the changes in bone tissue properties with osteoporosis in postmenopausal women, and the influence of menopause on the mechanical properties of bone remains obscure [22, 25–27]. Guo and Goldstein [22] measured the nanomechanical properties of trabecular bone in ovariectomy (OVX) rat using nanoindentation. They did not found distinct changes of the hardness or elastic modulus of trabeculae in the longitudinal section after OVX. The same conclusion was recently made for tibial trabecula by Lane et al. [28] and for transilial biopsies in healthy pre- and postmenopausal women by Polly et al. [29]. However, the hierarchically organized structure of bone has an irregular, yet optimized and elegant, arrangement and orientation of components, making the material heterogeneous and anisotropic [30]. The mechanical properties may vary with the cortical versus trabecular compartments, orientations, and types of bones [31]. Turner et al. [32] compared the elastic moduli between cortical and trabecular bone from the femoral midshaft and distal femur and found a pronounced difference in the elastic modulus of cortical bone in the transverse and longitudinal directions. Therefore, observations should be interpreted with cautions to compare the tissue-level mechanical properties of different types of bones. In these previous studies [22, 28, 29], the vertebral trabecular bone specimens were sectioned in only the longitudinal direction while the elastic modulus was measured in the transverse direction. Little data is available for bone sectioned in the cross-sectional direction.

Atomic force microscopy (AFM) provides a powerful tool to the mechanical properties of bone tissues at nanoscale [33]. It operates in the near field with a sharp probe by scanning the surface of the sample in a distance of a few angstroms. Compared with traditional optical microscope (TEM) and scanning electronic microscope (SEM), it enables characterization of three-dimensional (3D) surface morphology with minimal sample preparation and high resolution [28, 34, 35]. AFM has been widely used to visualize the bone matrix and to determine the spatial relationship between mineral and collagen and their morphology/topology as well [36, 37]. Furthermore, the combination of AFM and nanoindentation can characterize the structures and properties of both natural tissues and synthetic materials at the nanometer resolution [38, 39]. Nevertheless, the fragility of the nanosized components in bone has not been well understood.

The purpose of this study was to evaluate the effect of estrogen deprivation on the nano/microscale structural and mechanical properties of vertebral trabeculae in rats. We performed a series of experiments, including nanoindentation assessment of intrinsic material properties of trabeculae in the longitudinal direction, AFM characterization of trabecular nanostructures, and micro-computed tomography (*μ*CT) evaluation of trabecular microarchitecture.

## 2. Materials and Methods

### 2.1. Animals

Twenty 10-month-old Sprague-Dawley female rats, with body weight of 305 ± 10 g (mean ± SD), were provided by the Laboratory Animal Center of the Second Xiangya Hospital, Central South University, China. They were randomly assigned to two groups: ovariectomy (OVX) and sham-ovariectomy (SHAM), with 10 rats in each group. They were pair-fed, housed at 25°C with an alternating 12 h light/dark cycle, and allowed free access to water. At 15 weeks after operation, the rats were sacrificed. Their lumbar spine was dissected, wrapped in gauze soaked in normal saline, sealed in plastic bags, and stored at −70°C until measurements. All animal procedures were approved by our institutional animal care and use committee.

We chose 10-month-old rats because our previous study has shown that 10-month-old SD rats represent a good model of osteoporosis from estrogen deficiency [40, 41]. This study as well as our previous experiments [40, 41] shows no ovary atrophy in SHAM rats 10–15 months of age. The OVX rat models, especially young growing rat 3 months old, have been intensively investigated and widely used in the study of postmenopausal osteoporosis [42]. The young growing rats have main advantages, such as being relatively inexpensive to obtain and to maintain. However, the young growing rats have the great biologic disadvantages, such as continuously growing, not only in the primary spongiosa with open growth plate, but also with active periosteal bone apposition [43]. We previously have shown that cortical bone is not yet matured yet in rats until they become 7.5 months old [41]. In rats older than 10 months, the bone growth rate for the proximal tibial epiphysis is less than 3 *μ*m/day and stops after the age of 15 months. A female rat around 10 months of age has reached peak bone mass and can be manipulated to simulate clinical findings of postmenopausal osteoporosis [44].

### 2.2. Measurement of 2D Projectional DXA BMD

BMD was measured using a Hologic QDR 4500A dual-energy X-ray absorptiometry bone densitometer (Hologic, Bedford, MA, USA) under the conditions of 40 kVp and 100 kVp, with a scanning width of 18 cm and a velocity of 4.8 s/cm [5, 45]. The scans were analyzed with specific software for small animals. Quality-control scans were performed daily, using the manufacturer-supplied phantom, with a long term (2 years) root mean square coefficient of variation of 0.52%. The total body and vertebral BMD were measured in vivo, under general anesthesia, at baseline and again just before all rats were sacrificed. BMD of the dissected sixth lumbar vertebrae (L6) was determined after the animals were sacrificed.

### 2.3. Nanoindentation

A diamond blade saw (Buehler, Lake Bluff, IL, USA) was used to cut the center of the L6 vertebral body along the cross-sectional direction. The specimens were dehydrated in ascending grades of ethanol, with six changes from 70% to 100% and embedded in polymethyl methacrylate. The cutting surfaces of the embedded specimens were metallographically polished with silicon carbide abrasive papers with (1200, 800, and 600 grits). Then they were polished by using microcloths (Buehler, Lake Bluff, IL, USA) with fine grades of alumina powders. Finally, the specimens were cleaned in a distilled water ultrasonic bath for 10 min to remove surface debris [23]. The samples were tested dry immediately after polishing.

Nanoindentation is a testing technique commonly used to study the mechanical properties (e.g., elastic modulus, yield strength, and hardness) of materials at nanoscale [23, 24]. This technique can reduce the depth of indentation to the submicron range and extend the spatial resolution to about 1 nm [17, 23]. Oliver and Pharr proposed the theoretical basis of nanoindentation in 1992 [17]. The relationship between the contact stiffness and the elastic properties is

(1) $$\begin{array}{c} \frac{dP}{dh}=\beta \frac{2}{\sqrt{\pi }}\sqrt{A_{c}}E_{r}, \end{array}$$

where *β* is an empirical indenter shape factor and *A*_*c*_ is the projected area of the contact area. For the triangular pyramidal indenter, *β* = 1.034. The effective modulus *E*_*r*_ is given by

(2) $$\begin{array}{c} \frac{1}{E_{r}}=\frac{\left(1-{\nu_{s}}^{2}\right)}{E_{s}}+\frac{\left(1-{\nu_{i}}^{2}\right)}{E_{i}}, \end{array}$$

where *ν* is Poisson's ratio and the subscripts *s* and *i* refer to the sample and indenter, respectively. For bone, Poisson's ratio was assumed to be 0.3. The hardness is defined as the maximum load *P*_max_, divided by the contact area *A*_*c*_:

(3) $$\begin{array}{c} H=\frac{P_{max}}{A_{c}}. \end{array}$$

A fused silica sample, which exhibits elastic isotropy and has a small elastic modulus-hardness ratio, was used to calibrate the indenter tip shape function.

**Figure 1:**
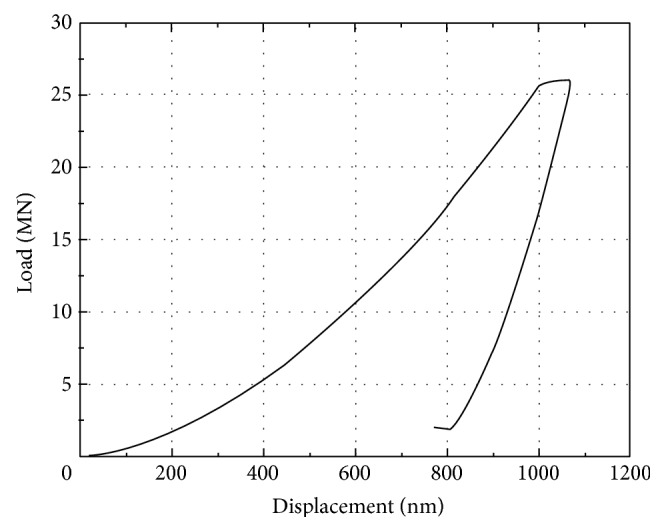
A representative load-displacement curve of nanoindentation.

**Figure 2:**
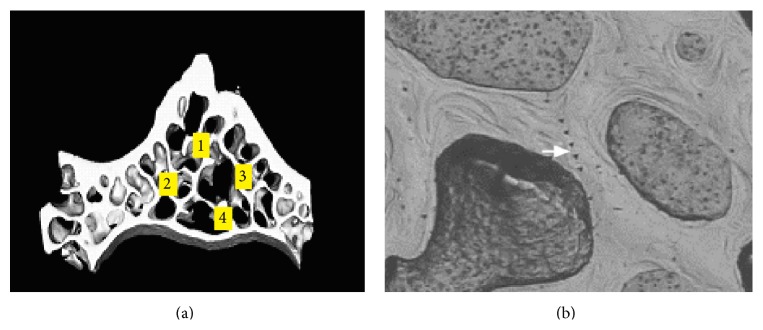
Schematic representation of the indent areas. On transversal slices of L6 vertebral body, nanoindentation test was performed randomly at four different regions (a). Five indentations were made for each site within 30–50 *μ*m (b).

All experiments were performed at room temperature (20°C). The Nanoindenter XP (MTS Systems Co., Oak Ridge, TN) was used, with force of 50 nN and displacement resolution of 0.01 nm [46].  Figure 1 presents a representative load-displacement curve of indentation. A permanent hardness impression was made by pressing the indenter into the specimen to a depth of 1000 nm at a constant indentation rate of 10 nm/s. There were two constant load holding periods in the whole process. First, at the peak load, the indenter was held for 100 s to minimize the effects of viscoelasticity and creep. The second constant load holding period, near the end of the test at 90% of the peak load, was used to establish the thermal drift rate in the machine and specimen for appropriate correction of the data [47, 48]. For each sample, four different trabeculae were measured to reduce random errors, as shown in Figure 2(a). The sites and directions of indentation were also randomly selected. Five indentations were made for each site within a 30~50 *μ*m region (Figure 2(b)) in the longitudinal direction of vertebral trabeculae, for a total of 20 indentations in the cross section of each sample in order to get the average results. The mean values and standard deviations (SDs) of hardness and elastic modulus were calculated by the arithmetic average of the obtained data under different depths, which were measured in the range of 400–900 nm displacements to avoid the effect of surface roughness.

### 2.4. Atomic Force Microscope (AFM)

At 24 h after indentation, the sample surface was cleaned with distilled water for 10 minutes, vacuum-dried at room temperature, and glued to metal disk for AFM (Digital Instruments, NanoScope IV/Dimension 3100; Santa Barbara, CA). The images of the indentations were recorded in air, at room temperature, at a scan rate of 1 Hz, in the tapping mode at the appropriate set point, and with a force constant of 40 N/m and resonant frequency of 300 kHz [36]. A depth profile, local morphological changes due to the indentation process, three-dimensional surface morphology, and roughness of the sample were recorded simultaneously.

### 2.5. Micro-Computed Tomography (*μ*CT) Morphometry [49]

The vertebral indentation specimens were immerged into deionized water and scanned using a Micro-CT specimen scanner (GE eXplore LocusSP Specimen Scanner; GE Healthcare Company, London, Canada). It is a cone-beam scanning system. The scanning protocol was 80 kV and 80 *μ*A, with an isotopic resolution of 6.5 × 6.5 × 6.5 *μ*m voxel size and an exposure time of 3 seconds per frame. The angle of increment around the sample was set to 0.4° resulting in 900 2D images. To increase the signal-to-noise ratio, each image was averaged over 4 X-ray projections. Both bright fields, that is, an X-ray projection with no object in the field of view, and dark fields, that is, an image acquired without any X-rays, were collected for correction of the acquisition images. A modified Feldkamp cone-beam algorithm was used to reconstruct 2D projections into 3-dimensional (3D) volume [50].

The original 3D image was displayed and analyzed with software (Microview ABA2.1.1; GE Healthcare Company, London, Canada). A fixed threshold value was used to binarize bone from other components.

**Figure 3:**
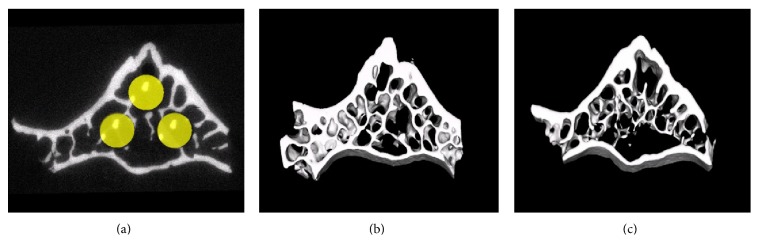
Micro-CT images of rat vertebral bodies. (a) Selection of the volume of interest (VOI) in the vertebral body for micro-CT analysis (yellow circles), with 3 VOIs in each vertebral specimen selected to contain the sites of indentations. Compared with sham operation (b), ovariectomy (c) results in pronounced trabecular deterioration of trabecular microstructure of the vertebral body.

The volume of interest (VOI) was defined as a cylindrical volume of 110 × 110 × 450 voxel size. Three VOIs in each vertebral specimen were selected to contain the sites of indentations (Figure 3(a)). The method described in the paper is unique, because the purpose of our study was to evaluate the effect of estrogen deficiency on the nano/microscale structural and mechanical properties of vertebral trabeculae in rats. It is critical that the region of each specimen examined by *μ*CT and AFM should contain the sites of indentations that were selected randomly. The method used in this study is different from other studies, because every experiment needs to customize the methodology to test its hypothesis [51–55].

Trabecular bone volume fraction (BV/TV), mean trabecular thickness (Tb.Th), trabecular separation (Tb.Sp), trabecular number (Tb.N), geometric degree of anisotropy (DA), connectivity density, structure model index (SMI), and trabecular volumetric BMD at both organ and tissue levels were determined.

### 2.6. Statistical Analysis

Statistical analysis was performed using SPSS 11.0 for windows statistical software (SPSS, Chicago, IL, USA). Independent-samples *t*-test was employed to compare the two groups after determining the normal distribution of the data. A probability (*P*) value of <0.05 was considered to be significant.

## 3. Results

### 3.1. Body Weight and 2D DXA BMD

Body weight and 2D projectional DXA BMD of OVX and SHAM rats were summarized in Table 1. 2D BMD of the L6 lumber vertebrae was significantly less in OVX than in SHAM, by −14% with in vitro measurement and by −12% with in vivo measurement. No statistically significant difference between SHAM and OVX was found in their body weights and total body BMD.

### 3.2. Micro-CT Analysis of Trabecular Microarchitecture

Table 2 summarizes the data of micro-CT analysis of vertebral trabecular bone. Volumetric trabecular BMD was significantly less in OVX rats than in SHAM rats, both at organ level by −27% and at tissue level by −12%. OVX induced a marked deterioration in microarchitecture of vertebral trabeculae (Figure 3). Trabecular BV/TV (−29%), Tb.Th (−14%), and TB.N (−17%) were significantly lower in OVX than in SHAM. Tb.Sp (19%) and SMI (91%) were significantly greater in OVX rats than in SHAM rats. No statistically significant difference in DA and connectivity density was found.

### 3.3. Nanoindentation Analysis of Material Properties

The trabecular elastic modulus was 24.609 ± 1.375 GPa for OVX and 25.275 ± 1.457 GPa for SHAM. The trabecular hardness was 1.085 ± 0.135 GPa for OVX and 1.098 ± 0.142 GPa for SHAM. No significant differences in the hardness and elastic modulus between the two groups were found.

### 3.4. AFM Images Analysis of Nanostructure at the Trabecular Surface

**Figure 4:**
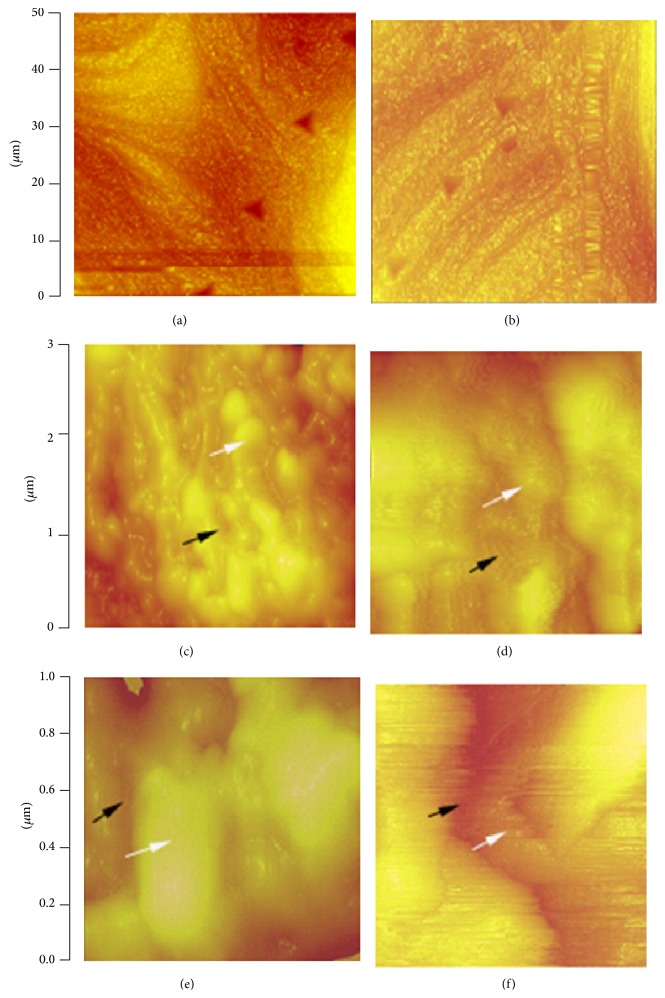
Typical atomic force microscopic topography of trabeculae of rat vertebral bodies of sham operated rats (left panel) and ovariectomized rats (right panel). The top 2 images (a, b) show unindented surface and indented impression. The middle 2 images demonstrate rough surface with many nodules closely packed to each other in sham operation (c), and relatively smooth surface characterized by some loosely packed large nodular structures in ovariectomized rat (d). The woven collagen fibrils of trabeculae from different group of animals have different directions. The interfibrillar space between collagen fibrils of a sham operated rat is more compacted (e), while ovariectomy results in larger size composite of collagen and mineral crystals (f).

Figure 4 shows typical AFM images of the unindented surfaces and indented impressions of vertebral trabeculae in SHAM and OVX rats. The difference in their surface morphologies can be clearly appreciated. The trabeculae of SHAM rats showed a rough surface with many nodules closely packed to each other, whereas OVX showed relatively smooth surface characterized by some loosely packed larger nodular structures (Figures 4(c) and 4(d)). The OVX bone showed larger composite of collagen and mineral crystals compared with the SHAM, while the interfibrillar space between collagen fibrils of SHAM was more compacted (Figures 4(e) and 4(f)). Little difference between OVX and SHAM was observed in the indentation impressions, indent depths, and residual area caused by plastic deformation. The average impression depth at 24 h after indentation was 172 nm.

## 4. Discussion

This study identified the structural features and mechanical properties of vertebral trabeculae in OVX rats at the micro- and nanoscales. Estrogen deprivation resulted in a dramatic deterioration in trabecular micro- and nanoarchitectures, while neither the hardness nor the elastic modulus of the vertebral trabeculae measured in longitudinal orientation was different from SHAM. Our observations of the cross sections of bone specimens were consistent with the previous data obtained from the longitudinal section [22, 28, 29]. The data indicate that the effect of estrogen deprivation on these particular intrinsic material properties seems to be insignificant, while effects on bone mineral density measures and on structure and geometry are conspicuous which is compatible with findings in our previous studies in several OVX animal models [3, 49]. Our study also shows that BMD changes after OVX were much greater in micro-CT 3D trabecular BMD at organ level than in 2D DXA BMD. This can be explained by greater loss in trabecular bone than in cortical bone in postmenopausal osteoporosis and by mask effects of relative unchanged cortical bone and end plates included in 2D DXA BMD. The more sensitive detection of bone loss induced by estrogen deprivation using CT than using DXA has been well documented, both in human and in animal models [43].

In this study, we used AFM to image the surface topography of trabeculae in OVX rats. While the composition and size of mineral crystals have been assessed in bone via different spectroscopic or X-ray methods [15, 56], their morphological evaluation through direct visualization in bone remains of particular importance [57]. We observed some bundles combining fibrillar collagen and embedded minerals. The collagen fibers and mineral crystals were packed in trabeculae and presented as nodular-like surfaces. OVX induced larger mineral crystals and looser interfibrillar space of collagen, which is consistent with a human study that large mineral grain size was found in the trabeculae of osteoporotic bone or age-related fractures [37]. Generally in various materials including pure hydroxyapatite, there is a size effect that the smaller the grain size, the higher the stiffness, the compressive and tensile strengths, and the fracture toughness [24].

The specimen of SHAM rats in our study showed a rough surface with many nodules closely packed to each other, whereas the surface of the bone sample of OVX rats was relatively smooth characterized by loosely packed large nodular structures. Milovanovic et al. [37] showed that, in contrast to young individuals, lower surface roughness and reduced topographical complexity in the elderly signify a decline in bone toughness. Estrogen-deficiency-induced changes in type-I collagen and collagen cross-linking in bone could be related to lower surface roughness and reduced toughness [58], which make the bone more brittle and susceptible to fracture. Previous studies showed that the physicochemical status of mineral crystals and bone matrix was significantly correlated with the mechanical properties of bone at the organ and material levels [59]. Interestingly, our nanoindentation tests of trabecula bone in OVX rats did not demonstrate a strong relationship between the nanoscale structural features and local tissue mechanical behavior, though our AFM study showed the particular contributions of the mineralized bone matrix from the morphological point of view. Further experimental studies are warranted to evaluate mechanical behavior at the interfibrillar level and to assess the mechanisms of grain enlargement.

The inconsistency between the negligible changes in the nanomechanical properties and the remarkable alterations of trabecular nano- and microarchitectures might be explained from the viewpoint of micromechanics. The negligible differences in the mechanical properties of bone suggest that estrogen deprivation mainly influences the spatial topologies of the constituent organic and mineral phases but not their nanomechanical properties. Though the nanomechanical properties of bone remain the same, changes in geometry and structures compromise macroscopic mechanical properties of bone. Previously reported two studies with changes in bone nanomechanical properties [27, 60] indicated that protein undernutrition associated with estrogen deficiency deteriorated bone tissue properties with improvement upon essential amino acids supplements. Thus, undernutrition rather than OVX was the main causation for the changes of material properties. Guo and Goldstein [22] attributed the unchanged nanomechanics to the unchanged density of trabeculae throughout adult life. Wang et al. [61] reported no difference in elastic moduli or hardness in human cancellous bone between normal and fracture groups. Polly et al. [29] used quasi-static and dynamic nanoindentation techniques to measure elastic and viscoelastic material properties of the trabecular bone and found no difference in bone intrinsic properties between healthy pre- and postmenopausal biopsies. We used AFM to record the residual area and depth profile of indents caused by plastic deformation and found no difference between the two groups, either in the residual area or in the average depth of indents. The material surface of OVX and SHAM bone had similar plastic properties, although different surface morphological characterizations were found. Our study is consistent with all these reports that bone nanomechanical properties identified by nanoindentation remain unchanged following OVX.

Small differences in the obtained elastic modulus values compared to previous published literature could be explained by the differences in the bone sample and the Poisson ratio of 0.3 used in this study. Oliver-Pharr method is based on an assumption that the sample is a perfect isotropic solid. However, bone is a complex multiscale anisotropic medium and is heterogeneous at the organ scale. Its mechanical properties depend on the cross-sectional and axial location [30]. Indentation modulus overestimated the elastic modulus in the directions with lower stiffness, that is, the radial and circumferential axes in long bone, and underestimated it in the direction with highest stiffness, that is, the superior-inferior direction [35]. Our study takes into account the anisotropic character of both the indenter and the sample but not all direction. Guo and Goldstein [22] found elastic modulus of 17.7 ± 4.0 GPa in transversely oriented vertebral trabeculae. Polly et al. [29] found 14.51 ± 3.39 GPa in human trabecular bone in the longitudinal direction. Our study analyzed longitudinally oriented trabeculae with 24.609 ± 1.375 GPa, which compares very favourably with previous nanoindentation studies. Rho and Pharr [48] found that samples of human trabecular bone, in the transverse direction, averaged 19.4 ± 2.3 GPa, while Brennan et al. [62] found an average modulus across the width of the trabeculae of 20.78 ± 2.4 GPa. Interestingly, all these studies agree that in trabeculae the elastic modulus is higher in the longitudinal compared to the transverse direction. Therefore, it is necessary to implement several measurements in different orientations.

Our study has several limitations. The duration of our observation was relatively short, which may also help explain no significant changes in the mechanical material properties of the trabeculae that still existed without enough time for resorbing and forming by remodeling, while newly built trabeculae after OVX would [63]. Quantitative measure of the size of principal topographic elements of the surface by AFM needs to be developed, while our assessment was quantitative. Poisson's ratio of bone might change among different samples and orientations, but in this study Poisson's ratio was assumed to be 0.3. Finally, the bone samples were performed in dry conditions, while a previous study has shown that the hydration testing condition had influence on nanoindentation testing [64]. Nevertheless, interspecimen comparisons in our study were reliable since all the specimens were subject to uniform processing and testing conditions.

## 5. Conclusion

In conclusion, estrogen deprivation after OVX in aged rat leads to dramatic deterioration in trabecular micro/nanoarchitectures, 3D volumetric BMD at both organ and tissue levels, and 2D DXA BMD as well, while the nanomechanical properties of trabecular bone remain unchanged. Further studies are warranted to develop quantitative measure of the size of principal topographic elements of the surface, to investigate the effects of mechanical loading or different treatments with various antiosteoporotic agents on the nanoscale intrinsic material properties, such as long-term administration of bisphosphonates that may result in subtrochanteric insufficiency fracture, to have better understanding of the composition, assembly, organization, and function of the fundamental building blocks of this amazing tissue.

## Figures and Tables

**Table 1:** Body weight and DXA measurements.

Parameters	SHAM (*n* = 10)	OVX (*n* = 10)	*P* value
Body weight (g)	332 ± 13	339 ± 9	0.739
Total BMD (mg/cm^2^)	170 ± 3	164 ± 2	0.068
L6 2D BMD in vivo (mg/cm^2^)	209 ± 6	179 ± 4^a^	0.000
L6 2D BMD2 in vitro (mg/cm^2^)	228 ± 6	201 ± 3^a^	0.001

Values are expressed as mean ± SD. ^a^ *P* < 0.01 versus SHAM.

**Table 2:** Trabecular volumetric BMD at organ and tissue levels and 3D microstructure evaluated by Micro-CT.

Parameters	SHAM (*n* = 9)	OVX (*n* = 9)	Difference (%)	*P* value
Organ BMD (mg/mm^3^)	478.5 ± 32.4	348.3 ± 41.2	−27.2^a^	0.002
Tissue BMD (mg/mm^3^)	820.1 ± 29.7	722.7 ± 36.5	−12.0^a^	0.001
BV/TV (%)	35.2 ± 7.3	25.6 ± 5.3	−28.6^a^	0.007
Tb.Th (*μ*m)	65 ± 7	56 ± 7	−13.8^a^	0.027
Tb.Sp (*μ*m)	177 ± 31	211 ± 47	19.2^a^	0.09
Tb.N (1/mm)	4.65 ± 0.59	3.86 ± 0.48	−17.0^a^	0.006
Structure model index (SMI)	1.02 ± 0.25	1.95 ± 0.31	91.2^a^	0.033
Degree of anisotropy (DA)	1.93 ± 0.29	2.04 ± 0.26	5.7	0.396
Connectivity density (1/mm)	35.6 ± 11.2	28.8 ± 9.8	−19.1	0.699

Values are expressed as mean ± SD. Difference: OVX versus SHAM, ^a^ *P* < 0.05.
